# Multiple, Pan-Enteric Perforation Secondary to Intestinal Tuberculosis

**DOI:** 10.1155/2015/318678

**Published:** 2015-12-20

**Authors:** Irfan Masood, Zain Majid, Ali Rafiq, Waqas Rind, Aisha Zia, Sajjad Raza

**Affiliations:** ^1^General Surgery, Civil Hospital Karachi, Karachi 74200, Pakistan; ^2^Dow University of Health Sciences, Karachi 74200, Pakistan; ^3^Sindh Institute of Urology & Transplantation, Karachi 74200, Pakistan; ^4^Thoracic & Cardiovascular Surgery, Cleveland Clinic Foundation, Cleveland, OH 44195, USA

## Abstract

Free perforation is one of the most feared complications of the intestinal tuberculosis. The terminal ileum is the most common site of perforation, while the majority of (90%) perforations are solitary. Herein, we describe a case of a 25-year-old male who presented with generalized peritonitis requiring an emergency exploratory laparotomy, which revealed pan-enteric perforation characterized by multiple perforations of the small bowel extending 10–15 cm from the DJ flexure up to the terminal ileum. The perforations were primarily closed, while 6–8 cm of the diseased terminal ileum was resected and the two ends were brought out as double-barreled ostomy. To the best of our knowledge, such an extensive tuberculous perforation of the small bowel has not been previously reported in the literature before.

## 1. Introduction

Intestinal tuberculosis is a disease of underdeveloped countries. However, the incidence is increasing in the Western countries due to increased influx of immigrants as well as increased incidence of HIV infection and increased use of immunosuppressive drugs. Perforation is an uncommon but life-threatening complication of intestinal TB, associated with high morbidity and mortality. We describe a patient who developed acute tuberculous pan-enteric perforation. Such a manifestation of intestinal TB has not been previously reported in the literature.

## 2. Case Presentation

A 25-year-old male presented to the emergency department with complaints of progressively worsening abdominal pain for the past 3 days, occurring on the background of fatigue and weight loss for the past 8 months.

**Figure 1:**
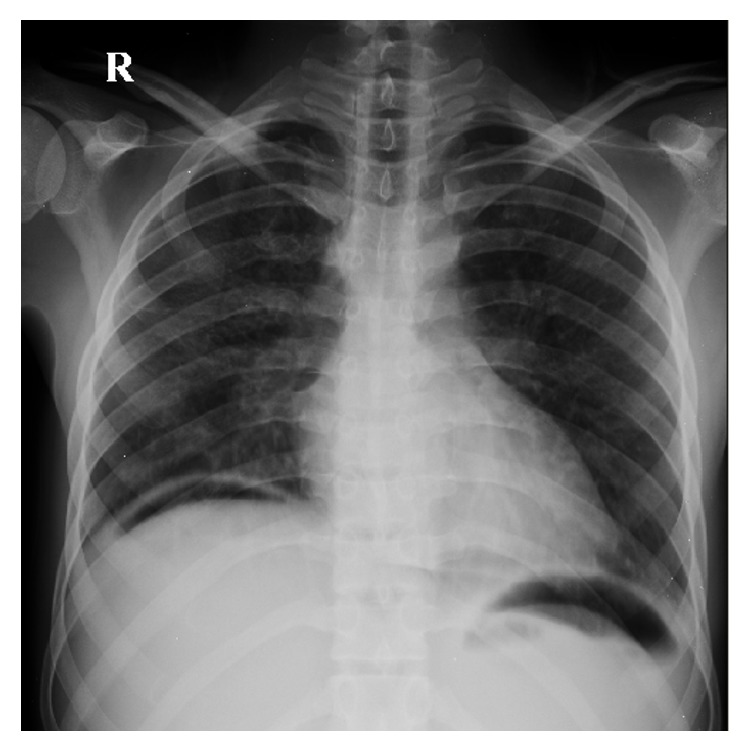
Erect CXR showing free air under the diaphragm.

The abdominal pain was continuous and severe in intensity and associated with multiple episodes of bilious vomiting, abdominal distention, and obstipation. On examination, the patient appeared to be confused and cachectic and was febrile (101°F), hypotensive (80/50 mmHg), and tachycardic (110 bpm). His abdomen was diffusely tender with board-like rigidity, while digital rectal examination (DRE) was unremarkable. His hematologic investigations revealed low hemoglobin (9 g/dL) and TLC of 15.4 × 10^6^. His electrolytes were within normal limits, but his serum albumin was 1.6 g/L. An erect chest X-ray showed free air under diaphragm (Figure 1).

**Figure 2:**
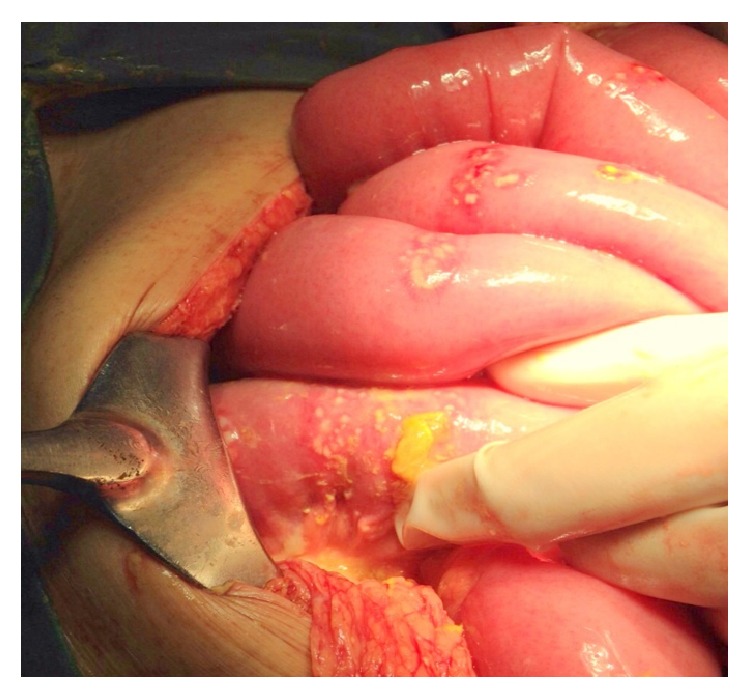
Multiple small bowel perforations.

**Figure 3:**
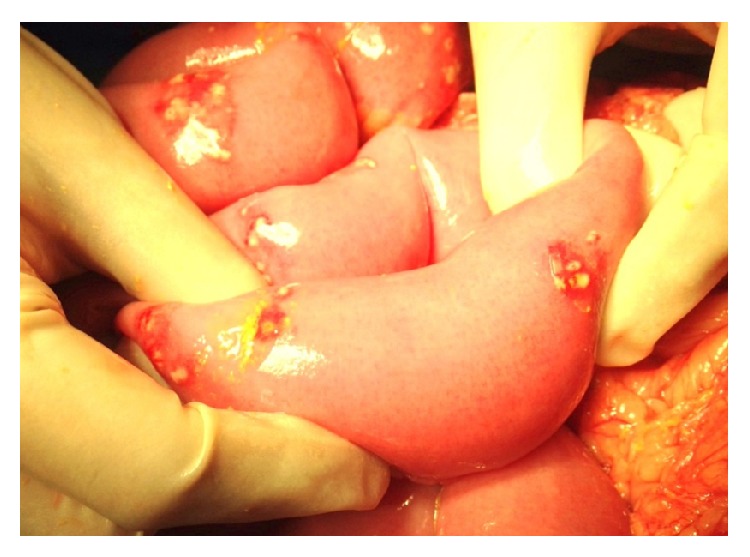
Each perforated site contained a confluence of pinpoint perforations.

After initial assessment a diagnosis of generalized peritonitis secondary to hollow viscus perforation was made. He underwent emergency laparotomy, with intraoperative findings of multiple small bowel perforations, starting 10–15 cm from duodenojejunal (DJ) junction, extending up to the terminal ileum (Figure 2). On closer examination, each diseased site had a confluence of pinpoint perforations (Figure 3).

**Figure 4:**
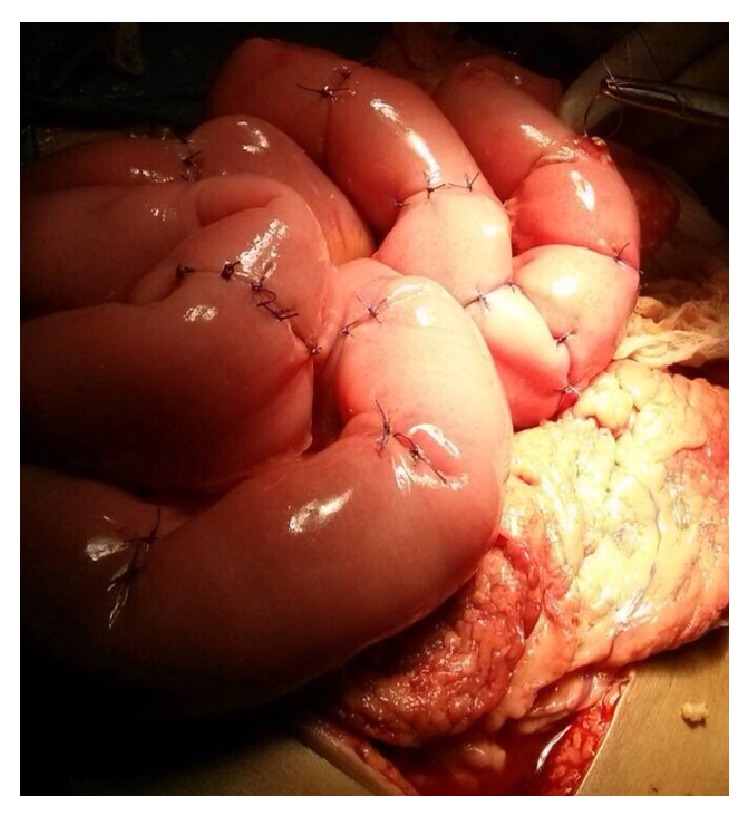
Primary closure of most of perforations.

Since the patient was hemodynamically unstable and severely wasted, such an extensive small bowel resection was not an option due to the high intraoperative and postoperative mortality risk. Therefore, a more conservative approach was selected. The perforations were primarily closed by Lambert sutures, using Vicryl 2-0 (Figure 4), while the perforations at the terminal ileum were resected and two ends were brought out as double-barreled ostomy.

He was subsequently started on antituberculous therapy based on clinical suspicion, and parenteral nutrition was also initiated. The histopathologic examination of the resected bowel specimen and the mesenteric lymph nodes was consistent with intestinal tuberculosis. The patient was closely watched for signs and symptoms of postoperative leak; however, he made a slow but progressive recovery and is currently being followed up in the clinics with ultimate plan of ileostomy closure.

## 3. Discussion

Intestinal TB is generally a disease of underdeveloped countries; however, due to an increase in the number of immigrants as well as HIV-infected patients, the incidence of intestinal TB is rising in the Western countries [1]. Only a minority of patients with pulmonary tuberculosis have intestinal involvement [2].

Free perforation is a complication of severe untreated disease, occurring in 1–15% of patients with intestinal TB [3, 4]. The most common site of intestinal perforation is the terminal ileum because of its high absorptive rate, close contact of the bacilli with mucosa, the presence of abundant lymphoid tissue, and being a region of physiologic stasis [5, 6]. In majority of cases (approximately 90%), only a single perforation is present [7], but multiple perforations at a single site such as the jejunum or the ileum have also been reported as well [1].

Dasgupta et al. studied tissue samples from patients of abdominal TB that had surgical intervention and perforation was seen in 80% of the cases, mostly of the terminal ileum and being solitary in nature, with epithelioid cell granuloma visible in lymph nodes and the intestinal tract [8]. However, in our case, there were multiple perforations 10–15 cm from DJ flexure up to the terminal ileum, with each site having a confluence of pinpoint perforations. Such an extensive perforation of the small bowel has not been previously reported in the literature.

Antituberculous chemotherapy is the mainstay of intestinal TB management; however, the duration of treatment is not uniform, ranging from 6 to 12 months [9]. Our patient is planned to have 9 months of antituberculous therapy, which was started on postoperative day 0 based on strong clinical suspicion and intraoperative findings. This regimen consists of a combination of drugs using isoniazid, rifampicin, pyrazinamide, and ethambutol. The histopathologic features associated with intestinal tuberculosis are important to distinguish it from other intra-abdominal pathologies, most importantly Crohn's disease. Macroscopically, features suggesting intestinal tuberculosis are bowel wall thickening, stricture formation, ulceration, and pseudopolyp formation. Strictures are short and concentric and have smooth outlines, while the ulcers are typically transverse with undermined edges. Microscopically, the hallmark features of intestinal tuberculosis are confluent, caseating granulomas that contain acid-fast bacilli surrounded by lymphoid cuff. These granulomas can be found in all the layers of the bowel wall and the regional lymph nodes [10].

Surgery is reserved for complication cases, such as obstruction, hemorrhage, perforation, abscess, and fistula formation. The most effective surgical therapy in perforated cases is resection of the affected segment with end to end anastomosis [1]. However, in our case, due to the extensive nature of the disease and cachexia, a more conservative approach was considered to be more appropriate. The majority of perforations were closed primarily, while 6–8 cm of the terminal ileum was resected and the two ends were taken out as double-barreled ostomy. The patient made a steady progress without any postoperative complications of anastomosis leak or intestinal obstruction and was subsequently discharged.

In summary, the case presented here is the first one to be reported in the literature that describes such an extensive perforation of almost the entire small bowel. Considering the fact that the incidence of intestinal TB is rising in both underdeveloped and Western countries, the aim of this study is to make the surgical community aware of such an atypical manifestation of the intestinal TB so that they are more prepared if such a case is encountered in the future.
